# A hitchhiker’s guide to an ISS experiment in under 9 months 

**DOI:** 10.1038/s41526-016-0003-7

**Published:** 2017-01-12

**Authors:** Andrei James Nadir

**Affiliations:** ISS Program, Applied Math, Science, and Engineering Institute, Valley Christian Schools, San Jose, CA USA

## Abstract

The International Space Station National Laboratory gives students a platform to conduct space-flight science experiments. To successfully take advantage of this opportunity, students and their mentors must have an understanding of how to develop and then conduct a science project on international space station within a school year. Many factors influence the speed in which a project progresses. The first step is to develop a science plan, including defining a hypothesis, developing science objectives, and defining a concept of operation for conducting the flight experiment. The next step is to translate the plan into well-defined requirements for payload development. The last step is a rapid development process. Included in this step is identifying problems early and negotiating appropriate trade-offs between science and implementation complexity. Organizing the team and keeping players motivated is an equally important task, as is employing the right mentors. The project team must understand the flight experiment infrastructure, which includes the international space station environment, payload resource requirements and available components, fail-safe operations, system logs, and payload data. Without this understanding, project development can be impacted, resulting in schedule delays, added costs, undiagnosed problems, and data misinterpretation. The information and processes for conducting low-cost, rapidly developed student-based international space station experiments are presented, including insight into the system operations, the development environment, effective team organization, and data analysis. The details are based on the Valley Christian Schools (VCS, San Jose, CA) fluidic density experiment and penicillin experiment, which were developed by 13- and 14-year-old students and flown on ISS.

## Introduction

An international space station (ISS) project is one of the best opportunities for students to gain an understanding of how to apply the book knowledge they gain in a classroom to a real-world application, while furthering the development of their technical and communication skills and education. Part of the challenge of putting an experiment aboard the ISS is defining and implementing an experiment to fit within the constraints of the ISS safety and technical requirements. This includes doing research^1^ on what others have done and looking into other published papers. For example, the density team researched several papers^2^ to know what to expect. But a more significant piece of the challenge is the development of the science hardware and software needed to interface the experiment into the systems aboard the ISS. In the past, this has been a very expensive and time-consuming endeavor. The Valley Christian Schools (VCS) (San Jose, CA) ISS Program developed a low-cost and rapid development plan and process to address these challenges, which includes standardized modular experiment hardware and software. In addition, VCS provides the science and payload development systems, student training, and much of the flight hardware and software in a pre-built package. Also, the VCS ISS Program manages the logistics to get an experiment aboard the ISS through partnering with a professional ISS implementation company, NanoRacks (Webster, TX), and National Aeronautics and Space Administration (NASA). Their support aids the Program projects through the NASA flight and safety approvals processes, payload manifest inputs, payload transport to and from the ISS, and the data telemetry receipt. Thus, this support transferred the high overhead cost and task efforts to professional implementers, which allowed the student project teams to focus on the science and development of their experiment payload.

### Project development

Developing an ISS experiment is both a science and engineering challenge. Science requires research, a hypothesis, an experiment plan, a test subject (biological, physical, etc), concept of operations, and determination of value added by microgravity. Determination of value is the amount of benefit that the resulting science return can have for furthering earth- or space-based research. Conducting a space-flight experiment on ISS requires defining all of the experiment requirements needed to properly conduct the experiment and determine if the experiment is compatible with available science flight hardware or if new hardware must be produced. The engineering challenge is to build or modify the science hardware to function on ISS in the microgravity environment, add back-up redundancy, and collect data, while ensuring that all science objectives are met. Both groups work together to conduct ground tests to verify hardware biocompatibility, hardware function, and the experiment operational plan.

ISS student experiments are a team effort. Therefore, the first process of any project is creating the project team, which includes having the right industry and academic mentors. The use of standard science and engineering and software industry design practices provides a structured method for identifying and completing tasks. The use of these practices significantly contributed to the successful implementation of fluidic density and the penicillin project, which are both outlined in this paper and were completed in 5 months. This paper discusses in detail some of the industry practices that are common to many of the VCS ISS experiments and provides recommendations on team structuring and responsibilities for efficient execution. There are other ways to accomplish the same outcomes, but this worked well for our student teams and is described in detail for two case studies, the fluidic density and penicillin experiments.

### VCS hardware development environment

**Fig. 1:**
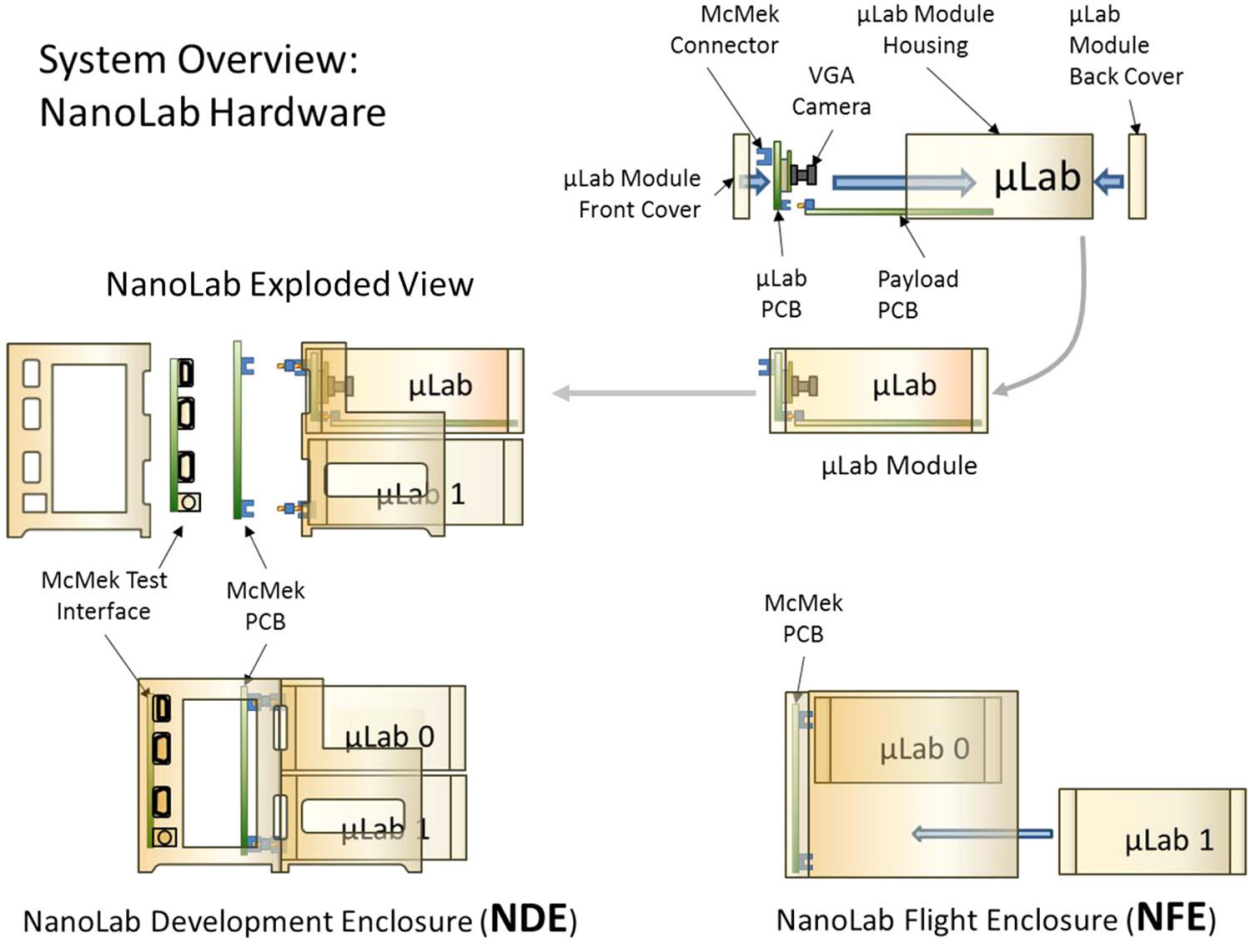
Development environment

Figure 1 is an overview of the hardware development environment. The VCS NanoLab is the master controller that interfaces between the science experiments and the ISS communication systems. The NanoLab holds up to four independent experiments, each of which is contained in a NanoLab subcomponent, VCS MicroLab (µLab).^3^

There are two configurations of VCS NanoLabs, one for experiment and µLab/engineering development (NanoLab development enclosure, NDE) and the flight unit (NanoLab flight enclosure, NFE). Both units monitor and control the µLabs with the main difference being that the NDE allows payload program access to the µLab. Programming access is not needed aboard the ISS and hence the µLab interface ports are removed. The NanoLab detects and interrogates the µLabs attached to it. µLabs do not initiate a transaction but must wait until it is polled by the NanoLab.

#### NanoLab

The NanoLab is the enclosure that houses the McMek control board. The McMek is used when referring to software operations, while NanoLab is used when referring to the physical enclosure. The words are often used interchangeably.

**Fig. 2:**
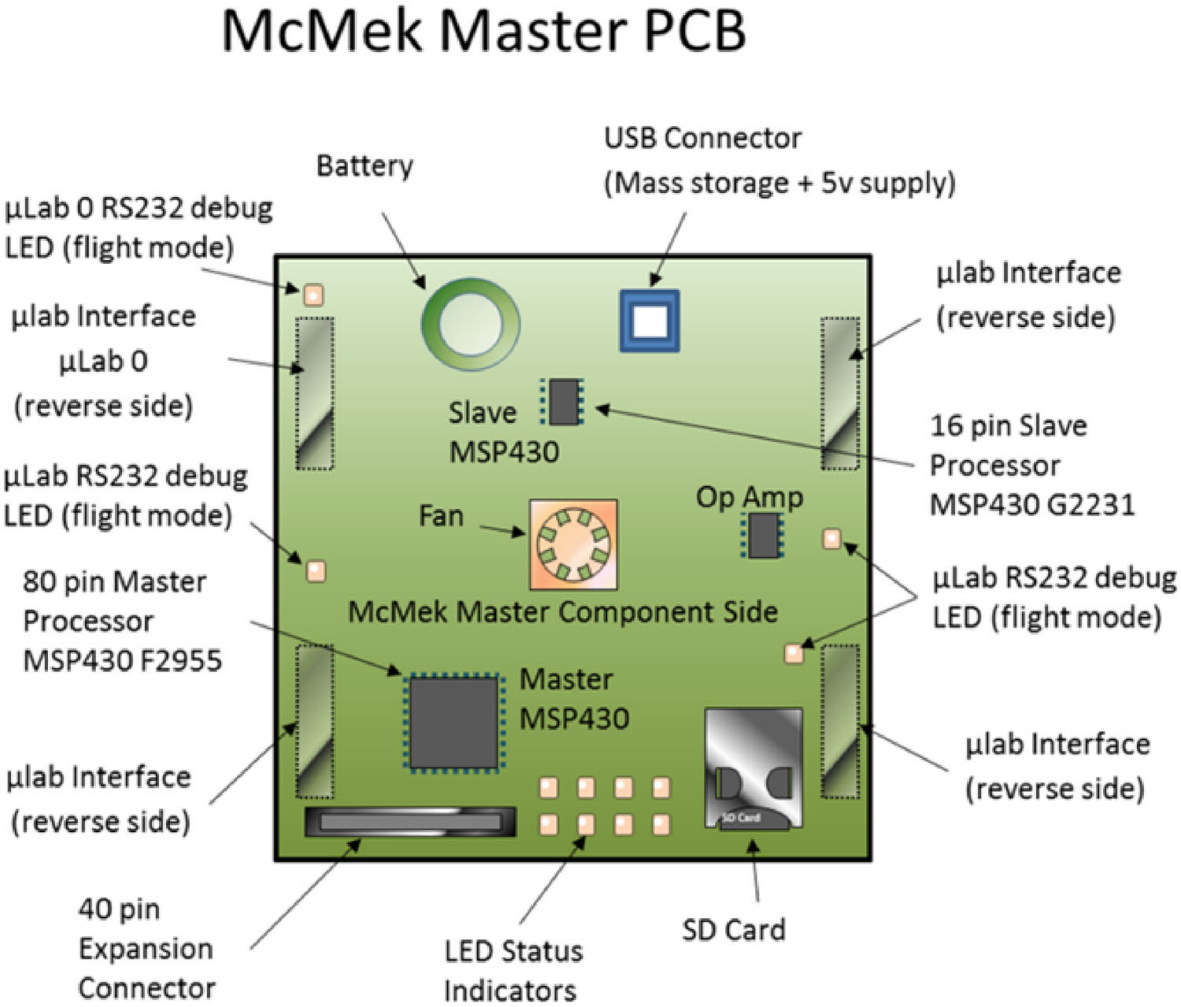
McMek master control board inside the NanoLab communication flow

The NanoLab (McMek) is the master controller that controls four independent µLabs and collects data from each one. It has a real-time clock, SD storage, two processors, backup battery, USB interface, two RS232 terminals, and status indicators (see Fig. 2).

**Fig. 3:**
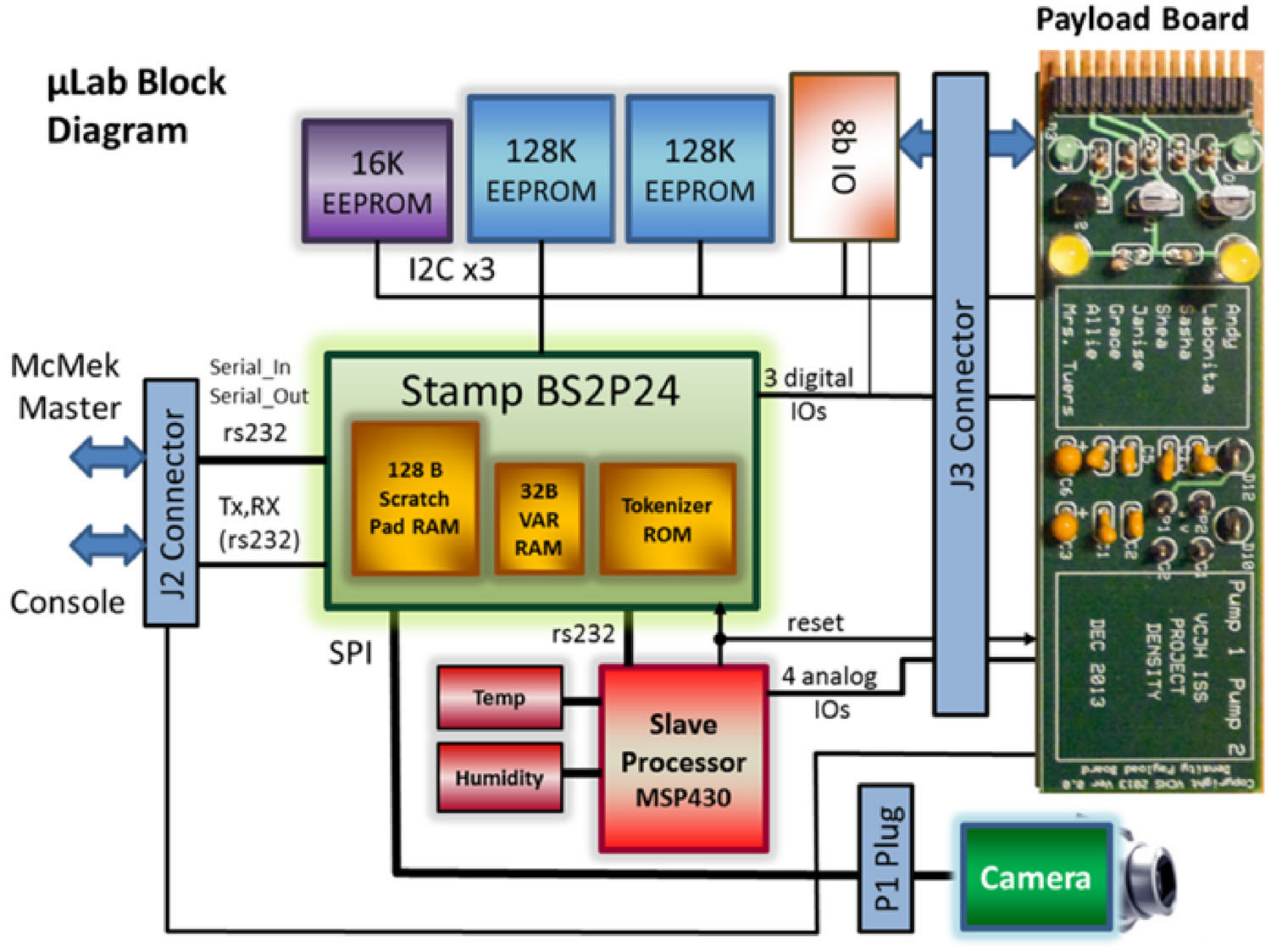
µLab block diagram

The McMek master processor holds the program that monitors and interrogates the µLabs. It also contains the USB memory interface to the SD Card that allows photos to be downloaded. The slave processor is a “watchdog” that monitors the master processor. If the master processor fails to respond within a given amount of time, it will be reset by the watchdog processor. This provides a “fail-safe” operation should the software go astray. Figure 3 illustrates the interfaces provided in the NDE. The McMek console is a RS232 terminal output of what the McMek board is doing. The stamp debug editor port interfaces the parallax stamp processor to its programming environment. For flight, the NFE does not carry the McMek Test Interface board along with COM port interfaces, leaving only the USB interface for communications.

The Payload Test Interface board shown in Fig. 3 is used to verify that the µLab is fully operational.

**Fig. 4:**
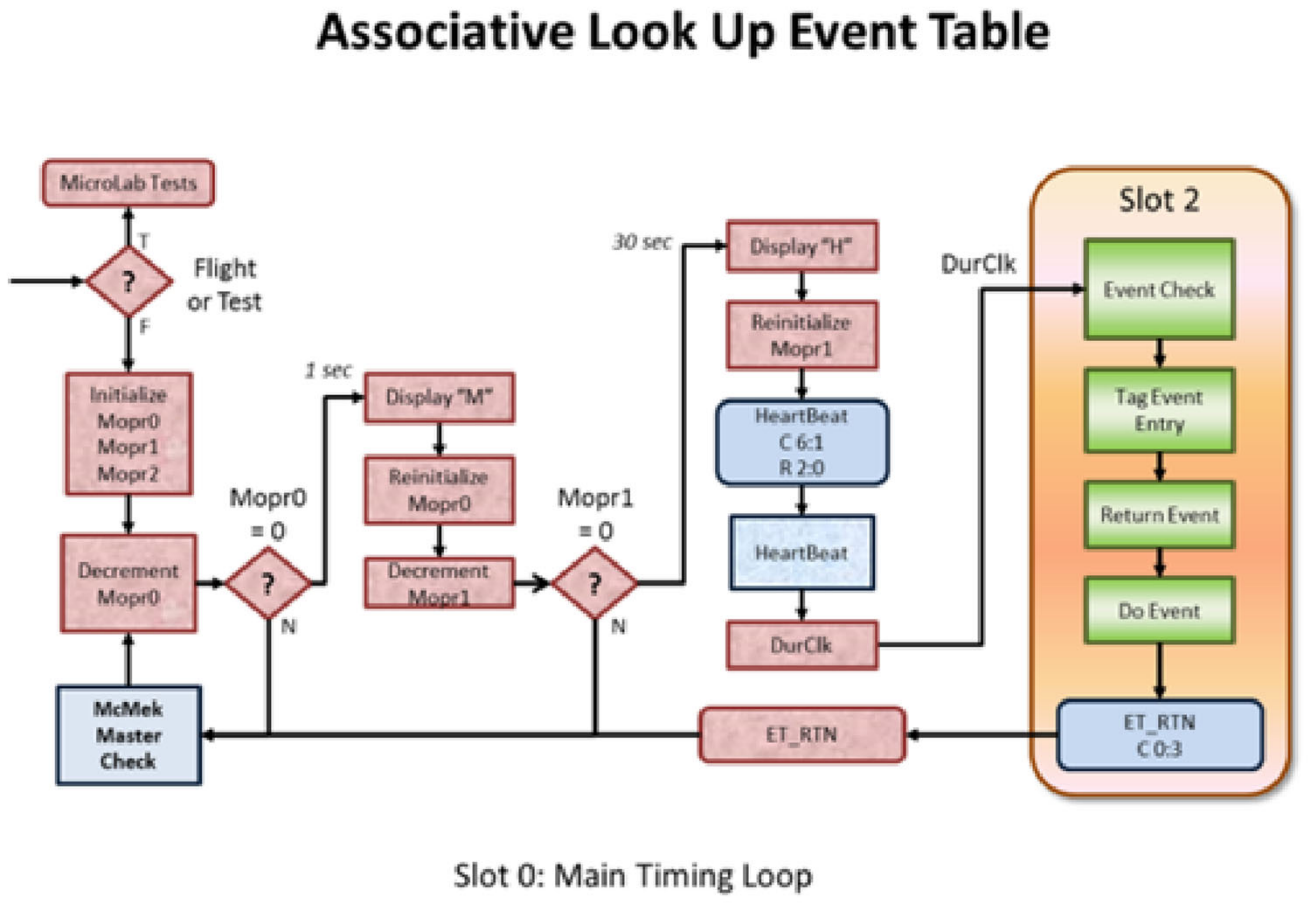
Basic BIOS template (*left*) and the Associate Event Table BIOS template (*right*)

The µLab is the enclosure that holds the experiment. It consists of the µLab board, a payload Printed Circuit Board (PCB), and experiment-specific components (water bags, pumps, valves, fluidic components, etc). Similar to the NanoLab, the µLab also has two processors, master and watchdog processors. The master processor (Parallax STAMP BS2P24) contains the software that runs the payload experiment. The watchdog processor will reset the master processor if it does not generate a “heartbeat” once every 45 s. Figure 4 is a block diagram of the µLab control board. The Stamp processor has a built-in Tokenizer, which supports the PBASIC programming language. The 16K Electronically Erasable Programable Read Only Memory (EEPROM) holds the PBASIC program and the two 128K EEPROMs can hold up to three photos to be uploaded to the Nanolab SD card.

#### µLab capabilities

- Four analog inputs with 10b A2D converter
- Payload temperature (Celsius)
- Payload humidity
- VGA camera with settable resolutions (480 × 680, 230 × 320) and quality
- 8B digital IO (open drain with 20K pullup)
- Three full-swing digital pins or I2C interface (Serial Data, Serial Clock) and one full-swing digital pin

#### µLab software: Basic Input Output System (BIOS)

The µLab comes with a BIOS ‘template’, which provides services, such as take photo, respond to McMek requests, perform heartbeats, and execute the payload program. Also, it provides system and payload testing. These templates are described below.

#### Common to all BIOS templates

- Programmed actions always occur immediately after a heartbeat.
- All templates initiate a heartbeat every 30 s. Failure to respond to a heartbeat results in a software reset to the µLab. The payload board needs to be designed to accommodate a µLab reset (i.e. pumps should be initialized to ‘off’ during the power-up reset cycle of the software).
- All templates respond to a McMek requests. Because the µLab may be engaged in doing something else, it is quite possible for it to miss a McMek request. A McMek request waits about 20 s for a response before moving on to interrogate the next µLab. However, after 30 consecutive McMek response failures, the McMek will power-down the entire payload and after 5 min power it back-up. This is also a fail-safe mechanism built into the system.

There are three templates available.

1. Basic: This is a template that supports all necessary functions to interface to the McMek and run the µLab. It comes with a pre-programmed main timing loop, which issues heartbeats, polls for McMek Requests, and takes a photo every 2 h. All templates are extensions of this one.
2. Index event table: This template replaces the photo per 2 h with a day–hour index table. The day–hour is used as an index into the table to return a user-defined byte when that day/hour is reached. This template is beneficial for controlling combinations of hardware resources at different times. For example, a plant experiment may wish to use combinations of colored Light Emitting Diode (LED)s along with Infra Red and Ultra Violet LEDs at different times. Defining the byte’s bit position to be on/off switches for different LEDs is very convenient and allows for many combinations.
3. Look-up event table (also called an associative look-up table): This is also an event table, but operates differently than the one above. Rather than use the day–hour as an address index, it looks up to see if there is an event for a particular day-hour-minute and if there is, it executes an associated event. It is the easiest to use the templates because it decouples the event actions from its timing. Events do not need to be in chronological order, actions are performed immediately (no decode of returned bytes) and it has minute-level resolution.

#### Power

Each µLab is allocated 100 mA. The processor consumes about 40 mA and an additional 60 mA is consumed when taking a photograph. When not taking a photograph, an experiment payload has about 60 mA. By properly managing resources (i.e. not doing a photograph while running a 40 mA pump or 60 mA vibrator motor), it is not too hard to meet the ISS power requirements.

### Payload system development milestones

#### Safety documents

One of the most important milestones is the early submission of the materials used by the experiment. Early development of NASA safety, hazards, and biomaterials documents enables identification or discovery of issues that can be addressed in a timely manner and reduce risk to delaying payload development work. A good example of this practice occurred during the fluidic density project and its use of watercolor pigments. Watercolor pigments were chosen due to easily discernable color contrasts and different density pigments. Cadmium Yellow, cadmium Red, and Indanthrene Blue were chosen, the first two being heavy-metal pigments and the blue being a light-weight dye. At the time, the safety document was one of the last items on the list to complete. As a result, the students did not know that cadmium metal becomes a dangerous aerosol in microgravity. The materials were immediately rejected by NASA, who returned the module for safe color substitutions. The student leader forwent attending his junior high graduation and then spent the day reloading the experiment with iron oxide red, titanium yellow, and ultramarine blue, so that the experiment could still be flown on its designated manifested flight to ISS. Since that incident, the VCS ISS Program now develops the safety documents as soon as the experiment is defined.

#### Ground tests

Ground tests consist of two components: (1) hardware component testing and (2) science experiment testing. The components of an experiment are tested before committing to a printed circuit board and other expensive resources. It is usually done using a breadboard. This is the first phase of the proof of concept and is an opportunity to determine if the hardware complexity will fit inside of the small enclosure of the µLab. It is also important to consistently make sure that all parts of the experiment are working without assumptions. Ground tests can be completed independently from the main project. For example, a student can test only the pump instead of waiting for the entire system to be designed. Then it is too late if a problem arises.

#### Flight tests

Before launch, it is imperative that at least one flight verification test has been successfully run. Flight tests are complete end-to-end emulations of the mission using flight hardware in its flight configuration carrying the science elements and all payload flight preparation procedures. Once a flight test starts, students do not interfere with it in any way, unless they intend to stop the test and start all over. It is important to eliminate any possible problems by finding them with a flight test. Any issues that are not fixed on ground will cause major problems in space. A key factor of this test is the demonstration that the readiness of the payload is certified for flight. Changes that are identified after this test is completed may require repeating the flight verification tests. Therefore, it is important that this test is run at least 2–3 months before the payload is turned over to the ISS integration team.

## Experiment design and execution

The project infrastructure of the VCS ISS program has removed many obstacles to the rapid development of ISS student experiments. One key factor is a structured, standardized method for starting a project. The VCS ISS Program uses a set of questions (see Table 1) that helps the students organize their thoughts and ideas before launching into the development of the experiment and ideas in order to eliminate wasting time in unproductive science and engineering discussions and evaluations.

**Table 1:** Pre-experiment questions

Y/N	Questions to ask before starting the ISS experiment
	Is the science well understood and implementable within the constraints of the system and available timeframe?
	Can the experiment be implemented with off the shelf components or components readily available from VCS?
	Has the influence of microgravity been clearly articulated and understood?
	Does the research extend or confirm the findings of a previous experiment?
	Does the experiment have redundancy in case there is a component failure?
	Are the expected results measurable with the available resources?
	Are biological components such as cells and cultures readily available and affordable?
	Are the materials safe should they escape containment? Do you have Material Safety Data Sheet for each one?
	Have your materials come from a reputable and verifiable source?

Answering ‘no’ to any of the above questions could entail significant amount of effort, out of scope costs, unexpected delays, and might warrant rethinking the nature of the experiment being considered.

### Best-known design practices

The VCS ISS Program has developed a set of lessons learned and best practices over the year. A compilation of best-known design practices to enable a fast and efficient execution of an ISS experiment are presented below. These practices also come directly from the practices used in industry.

#### Organizing teams

A well-functioning team can overcome many obstacles. One of the most important ingredients is that the team members all have the same desire and passion for the experiment. Also, the team members make it a priority to be inclusive and appreciate communication. Choosing teams based on their academic achievement in the classroom is usually not the most effective method for putting together a cohesive team and for identifying problem solvers and innovators.

Participating in an ISS flight experiment is a very large time commitment due to not only the internal project schedule but also having to meet NASA and implementation partner schedules. The rocket will fly with or without the student experiment, so the experiment must be ready on time. Students interested in participating need to evaluate all their activities, commitments, and priorities before signing up. Overbooked team members or those with other huge commitments such as robotics, sports, dance, orchestra, or band are unlikely to have the time required to commitment to the team, especially in times when the project is not progressing smoothly. Finally, students who only want to participate to check off an item on their college resume tend to be less interested in actively participating, which adds more work to other team members. Experience over time has allowed the VCS ISS program the ability to identify candidates who show characteristics of a contributing or noncontributing team member (Table 2).

**Table 2:** Team members

Contibuting team member
Strong interest or passion for science and/or engineering and/or strong desire to learn
Able and willing to commit time to the project; pro-active
Innovative and can think out of the box
Willing to do the work needed to research information
Willing to take on challenges – inside or outside their comfort zone
Good communication skills and works well with others
ISS Project is their only or top priority commitment
Provides both intellectual and technical contributions to the project
Able to take constructive criticism and learn from failure
Noncontibuting team member
Only wants a data point on their college application
Uninterested in science and engineering with no possibility of developing interest
Other higher priority commitments and time-sink activities
Willing to put in the minimum effort but no more
Not a good team player
Seeks personal recognition over team work
History or propensity to ignore projects or miss team meetings and activities
Must have their own ideas, concepts, etc selected

#### Function as an engineering and software industry team

A working team in industry is not the same as student functioning in a classroom. In the classroom, there is a premium placed on right answers and essays that conform to specific formats or curriculum-defined standards. Also, students focus on individual success and achievement because that success is important for college applications. However, in industry there is no one right answer. Also, priority is put on the success of teams and individual contributions are seen in context to the success of the team project. All tasks are equally important, and each one is necessary but may result in different experiences being received by each team member either due to past experience or due to capabilities. To overcome this difference in experience, the VCS mentor for the junior high team returned to the classroom environment after the project was delivered and brought everyone up to speed on programming, electronics, and hardware design. Other mechanisms for providing an equal learning experience are team meetings.

In industry “rapid prototyping” processes are the norm, while in the classroom the “fear of making a mistake” dominates the effort. The fear of making a mistake reduces freely creative thinking and stifles the intellectual creative process due to a need to always get the right answer first time. This type of fear can negatively impact efforts to go from concept to design in a timely manner, while investigating and testing many different design concepts along the way. Rapid prototyping is an engineering/software industry concept that “throws something together as fast as possible” to test concepts and predictions, address known unknowns, discovery of unknowns, and define the boundary conditions of the components. The early knowledge gained from rapid prototyping allows time for innovation and reduces the emotional commitment to preserve work from going to waste. Most importantly, it allows for all reasonable ideas and concepts to be addressed in a formal manner, which reduces the risk to missing or eliminating a critical element.

### Rapid prototyping leads to early discovery and innovation

Fluidic density and the penicillin experiments were the first to use rapid prototyping at VCS. Since then the practice of rapid prototyping has spread to the high-school experiments with similar successful results.

**Fig. 5:**
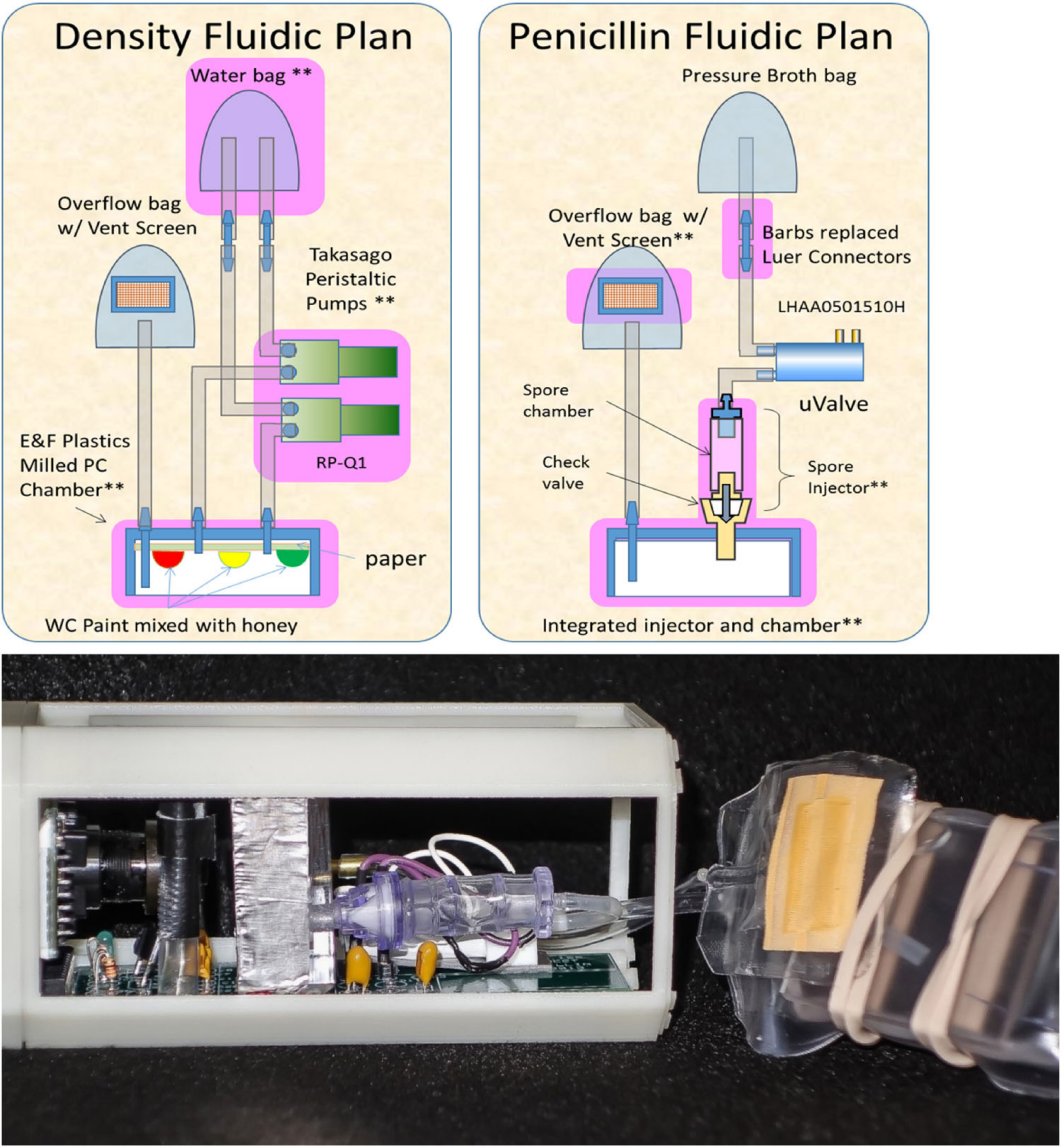
Rapid prototyping innovations of fluidic density and penicillin experiments

The fluidic density project goal was to mix watercolors of different densities in the ISS microgravity environment to examine if Van der waal forces would dominate mixing. Their first attempt was to use the traditional pressure bag and valve to push liquid watercolors into an observation chamber and photograph the results. Using rapid prototyping the team quickly discovered that pressurized liquids would create turbulence, which would mix the colors too quickly to be seen on camera. In addition, the team discovered that they did not have room for three separate pressurized bags and valves (Fig. 5).

**Fig. 6:**
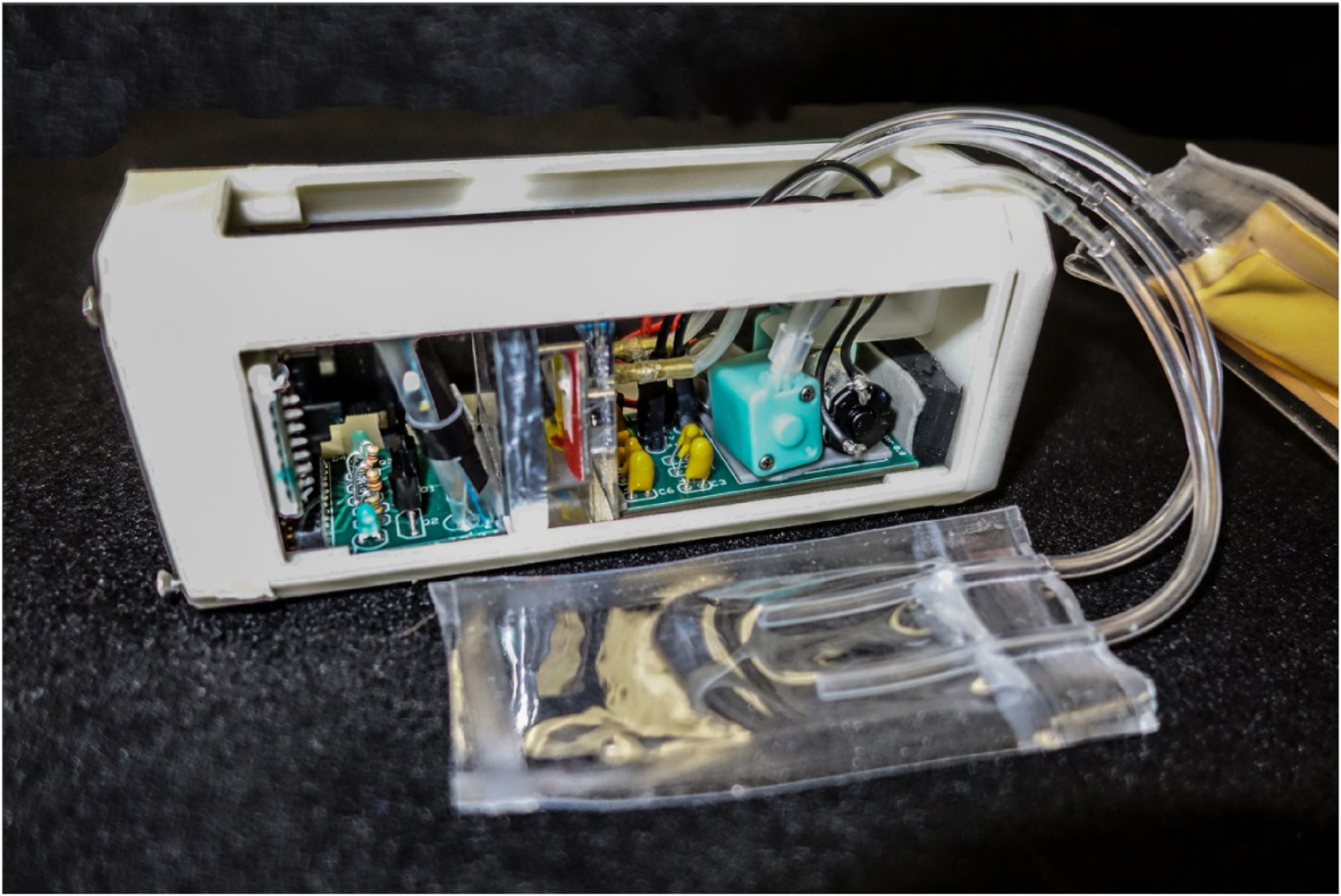
Fluidic density showing the innovated polycarbonate chamber, low profile water bag, vented pressure bag, redundant peristaltic pumps, and anti-glare jackets, which are highlighted in *purple* in Fig. 5

The early identification of technical issues and discovery of other problems led to a new innovation for using 40 mA peristaltic pumps to replace the 70 mA valves and to eliminate space-consuming pressurized bags. This innovation led to cross-pollinating other projects through the development of the spore injector for the penicillin project to hydrate and mix penicillin spores, the development of large polycarbonate observation chambers, and the development of low profile water bags and vented overflow bags. Figure 6 illustrates the shared commonality of the fluidic density and penicillin projects (the areas highlighted in lavender are the junior high innovations that crossed over into VC high-school projects and other VCS ISS Program companion high schools).

In summary, rapid prototyping led to the early discovery of many problems, which in turn enabled identification of innovative solutions for both the fluidic density and penicillin projects. The rapid prototyping was a key factor in the ability of both teams to have their flight projects ready in 5 months to the payload-delivery schedule for launch.

### Team organization and roles

#### Team leader and co-lead (communicator)

The team leader and co-lead should be selected by staff/mentors who know the maturity and leadership abilities of individual students. The best leaders are those who are well-organized, even-tempered, pro-active in engaging everyone, inclusive, good communicators, especially in constructive criticism, and encourages all team members to take ownership of their tasks and be pro-active problem solvers. Team leaders are good technically and can present information concisely. Also, a team leader recognizes that the project is a team effort and not their own project, and that participation on an ISS project is as much an educational experience as it is a commitment. Should an issue arise with someone’s participation, the leader should be capable of constructively confronting that team member, and, if necessary, work with mentors to resolve on going issues.

**Fig. 7:**
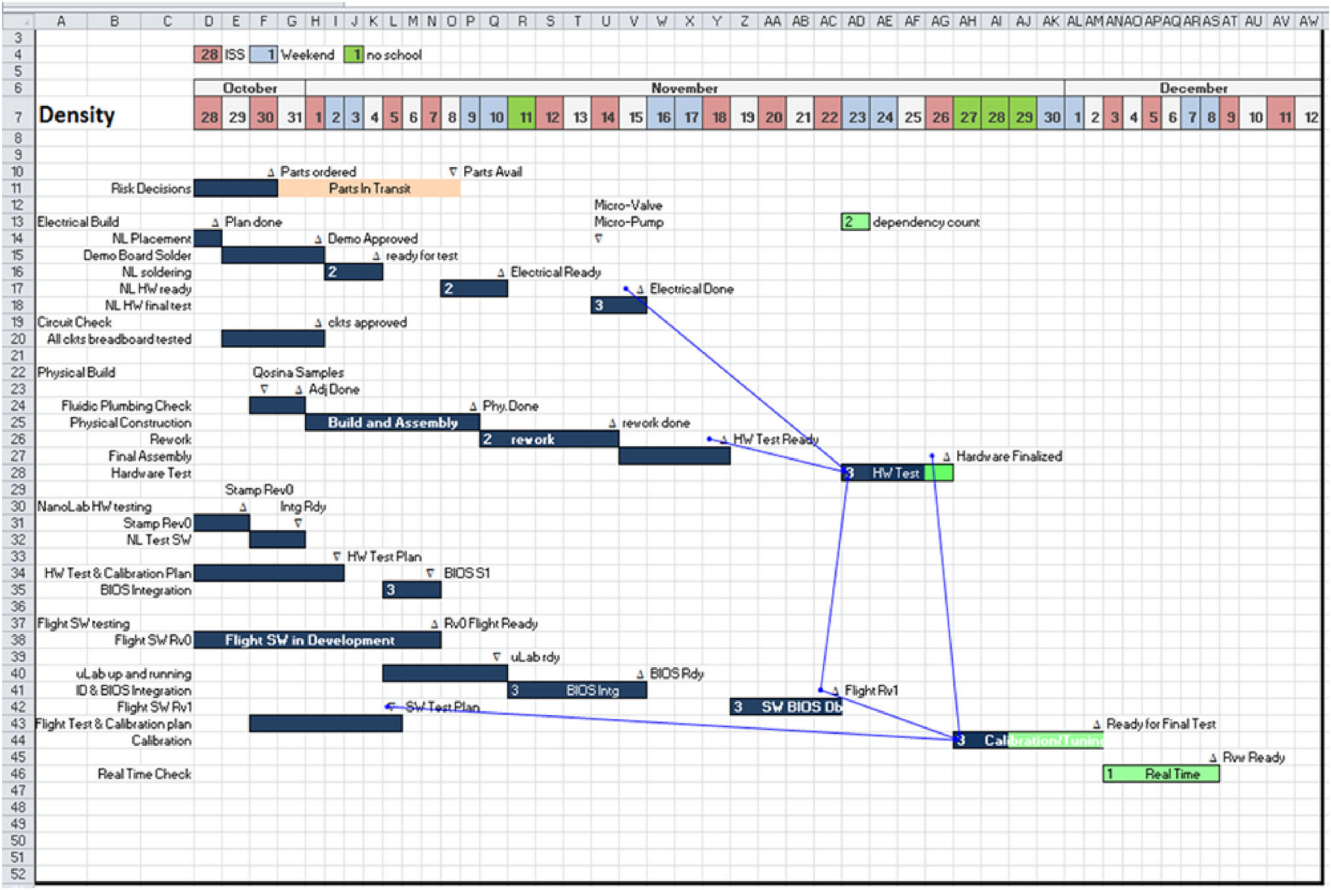
Example Gantt project schedule used by the density team leader showing dependencies between activities

**Fig. 8:**
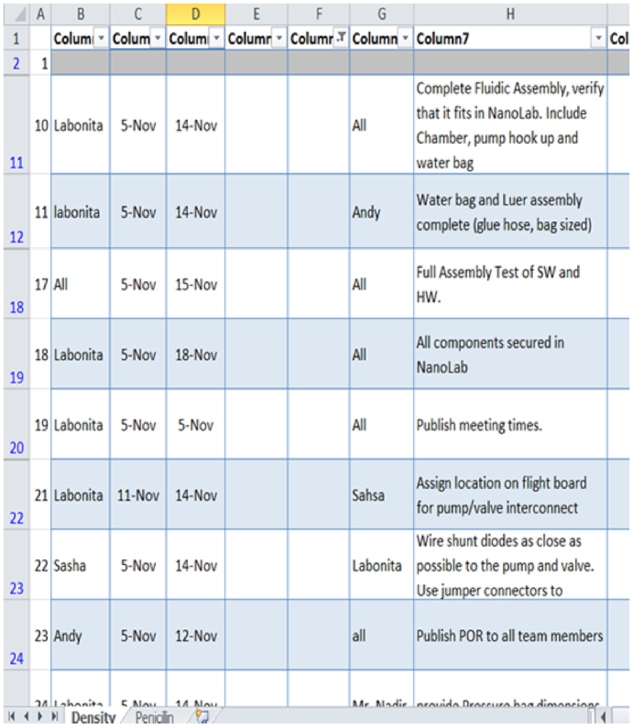
Example action item list maintained by fluidic density co-leader

The team leader’s primary role is to communicate, negotiate, judge effort levels, and should be a person who is willing to make hard decisions for the betterment of the team and project regardless of personal outcomes. The team leader facilitates team member interactions and monitors progress of the project, including its schedule and resource needs. The leader communicates the “what”, negotiates the “when” but does not tell the “how” defining “how” is the responsibility of the team member who owns the task. The leader can ask “how” and negotiate effort but cannot dictate it. However, if necessary, the team leader can bring the team together to brainstorm ideas and the “how” if the team member needs help (Fig. 7 and Fig. 8).

The role of co-lead is to communicate the intent and “spirit” of the task intent and to follow up regularly with team members to make sure that they understand what is expected and when it is needed. In addition, the co-lead closely participated in the development of the schedule and milestone so that (1) they would understand the intent and (2) could act as lead should the leader be absent that day.

In the past, less effective methods were used to communicate tasks. One method was to simply discuss the tasks at the beginning of each team meeting. This discussion would cover the action items of all students for that day. It was time-consuming and often not followed up until the following team meeting. Team members were often embarrassed to ask for task clarification in front of others or thought they understood intent (but did not). Tasks that had to be completed between team meetings were often forgotten.

Another method that was used in the past was to communicate through email and texting. This approach also proved to be ineffective because team members would often not respond to the messages. Some task owners claimed that they did not receive an email (i.e. caught in spam folders) or text message. Of those that did respond, some would craft the responses to what they thought the co-lead wanted to hear or fear to admit failure (a side effect of the academic environment where a failure is unacceptable). The end result was that the task was not done on time. In addition, email/texting was not effective in soliciting feedback in a timely fashion on the owner’s understanding of the task.

Eventually, the team leadership evolved to an industry model (our mentors were from industry), where the discovery of progress was done through a “one on one”. This was owned by the co-lead and was done, either in a face-to-face discussion or alternatively through a phone call. This spontaneous form of communication allowed the co-lead to solicit the task owner to “playback” their understanding of their current task, correct misunderstandings, to seek clarification of progress, and to catch subtle hints that there might be a problem arising, “text and forget” messaging. In summary, task owners always had to confirm their understanding of their task and could ask questions if they still had issues.

After the “one-on one” progress was recorded and maintained on an action list (another industry method). The co-lead was responsible for recording progress and maintaining the action list. The co-lead was responsible to review the action list progress and issues at the start of each meeting. To prevent the action list from usurping the tasks on the project planning schedule, the scope of the action list was limited to a 2-week “rolling window”. In other words, the action list could be viewed as a “magnifying glass” enlargement of the tasks on the overall project plan.

Despite owning the AR list, the co-lead does not make the action list items. Those are created either by the team leader or by the system architect. The lead remained responsible for rebalancing resources and negotiating task efforts when the project plan needed to change to accommodate and resolve issues.

The job of the co-lead allowed the leader to focus on the overall implementation, rebalancing resources to address problems and to identify issues that crossed task owners. As mentioned earlier, the co-lead needs to be able to step in if the leader is absent, and therefore had to be involved in the project planning process.

Finally, although the leader and co-leader are viewed as the primary representatives of the team, they must take credit for work done by their team members. They need to push the responsible team member to present their work and to take the credit they deserve for the work they did.

Another responsibility of the team leader is to recognize when a design passes through a development phase and into the completion phase of the design. Here the leader must restrain “creeping elegance”, which can cause the schedule to diverge instead of converge. Usually this is done through another industry practice called an engineering change order (ECO). The task leader is responsible for deciding when the project goes onto ECO process. When it does, any change, before it can be made, has to be pre-approved by both the lead and co-lead. The ECO process can be thought of locking parts of the design into a vault to force design convergence (Table 3).

**Table 3:** Team leader tools

Tools	What it’s for
Gantt Chart	High-level bar schedule of the development flow and high-level phases of design completion. Each phase has a measurable milestone (deliverable) as its end point, who is the customer for its deliverables, assigned owners, and due dates. The Gantt Chart is reviewed as design phase reach their milestones or when changes are made. See Fig. 7
Action lists	Short duration actions that need to be done to keep the project on schedule. These actions are usually not listed on the Gantt chart because they are short term in nature (about 2–3 days on the average). Every action list has a measurable deliverable, an assigned owner, and a due date. Action lists are reviewed regularly at the beginning of each meeting. See Fig. 8
ECO	ECO is the process of converging the design as it nears completion. When a design nears completion, the leader puts the project on ECO which effectively locks the design from further changes without his/her authorization. Failure to do so will result in creeping elegance and schedule slips

### System architect

The system architect is responsible for understanding the issues between design silo (software, hardware, electrical, science, etc.) and to make tradeoffs between them. An important role of the system architect is to make sure that the science is implementable within the confines of the µLab environment and, if not, to drive changes in the science, the hardware, or both so that the experiment is implementable in the allocated timeframe. Also, the system architecture is responsible for capturing and monitoring all problems and hardware and science nonconformities that occur.

### Electrical team

The electrical team has a worker and a leader. The leader’s responsibility is to communicate with the team leader and the system architect about his or her team’s progress and what they need completed by other teams for them to continue. The electrical leader partitions the work that needs to be done and participates in the effort. One person is usually assigned to develop the schematics and lay out the PCB while the other builds the rapid prototype board and tests it functionality.

### Hardware team

Much like the electrical team, the hardware team has a leader and one or two workers. The leader is responsible for communication with the system architect and the team leader. The team leader creates a scaled block diagram with the help of other team members and makes sure that everything fits, is drawn to scale, and that hardware functions as intended. This team builds the hardware for the rapid prototype as well as the final flight unit hardware. They are also responsible for innovating solutions to the mechanical and space problems that arise.

### Software team

The software team is different from the other teams. The size of this team will range with the difficulty of the code. It still has a leader that reports to the team leader and workers.

### Science team

The role of the science team is to research, identify and communicate with professional scientists (advisors), lead the science experiment design and requirements definition, and do the wetlab science tests. Additionally, they need to make sure that the goal of the experiment is something that can be completed with the time constraints and are being met by the engineering and science team tasks.

### Quality assurance (QA)

QA is responsible for providing an independent assessment and control of the processes and procedures of the project. It may be assumed that the team member who designed the component (software, hardware, circuits, etc) is the best person to test it and confirm all work was done to drawing and procedure. However, this person is too close to the work and cannot be authorized to check his/her own work. In industry, a different individual not associated with the specific task is responsible for testing the system and verifying the work. This is because these individuals do not have the same assumptions as the original designer and do not have the same emotional attachment to the design or work. These individuals look at the design from a nonbiased and neutral perspective. For example, in biology projects, this includes often overlooked issues such as noncompliance with material toxicity and biocompatibility within the observation chamber that results in killing the specimen. It also includes checking for chamber leaks, broken tubes, software anomalies, and faulty or poor workmanship. It is imperative that the individuals on the testing team do not check their own parts of the project.

#### Mentors

Having an industry mentor experienced at project management is key to having a successful team. Mentors with science or software along with hardware and/or electrical knowledge are ideal but rare. To address this issue, many schools have several mentors, each having a skill set in one or more of design disciplines of project management, software, electrical and hardware (Table 4).

**Table 4:** Engineering vs. classroom environments

Engineering is not the same as classroom—not everyone gets the same experience
Create a Gantt chart schedule, track regularly and adjust
Use action lists to track short-term tasks, not the overall schedule or Gantt chart
Set expectations and regularly show progress and owners
Use rapid prototyping for early problem discovery and Innovation
Hold individuals accountable
Push team members outside of their comfort zones
Too many team members is as bad as too few
Use ECO to converge the design

#### Students and team members

Below are words of advice to prospect team members considering joining an ISS development team:

- You really have to want to make a difference
  - Design, solve problems and innovate
  - Do not let your friends “pull you back” and do not come for the “bragging rights”
  - Do not consider participation as only a item to check off on your college application
  - Test your capabilities and education and learn more about what you may want in a career
- Come to learn how to function as those in industry
  - The learnings are applicable to all industries
  - Learn about team work in a real-world applications setting
- Not everyone can be (or wants to be) leader
  - The leader is a “hostage” between the individual players and the schedule
  - Leaders are not technical know-it-alls. Their job is to discover obstacles and motivate
  - Trains the less experienced player and doesn’t cast shadows on others
- Your most important learnings are from industry and academic experts and their experience.
  - But do the research to find out what others did before asking the mentor
  - Learn from industry and academic experts
- Do not be afraid to make mistakes
  - You learn more from a mistake
  - Mistakes are opportunities for innovation
- The job doesn’t end with the bell—Be prepared to put in the extra hours or the weekend
- This is not a test
  - Try to solve the problem on your own. This is where you grow. But if you are floundering seek help

#### Design summary

This paper has presented the ISS development support infrastructure and how to organize a team to quickly implement an experiment^4^ to go aboard the ISS in 6 months based on the VCS ISS Program years of experience conducting student-led science and engineering projects. The infrastructure services was briefly described and shown to do most of the heavy lifting to interface an experiment to systems aboard the ISS. It is available for low cost through VCS. In addition, the paper described one way to organize an effective team along with brief descriptions of roles and responsibilities. And it was shown that with the right industry and academic mentors the path to the ISS is not as daunting as it might first appear.
