# Erratum: A hitchhiker’s guide to an ISS experiment in under 9 months

**DOI:** 10.1038/s41526-017-0017-9

**Published:** 2017-08-02

**Authors:** Andrei James Nadir

**Affiliations:** ISS Program, Applied Math, Science, and Engineering Institute, Valley Christian Schools, San Jose, CA USA

**Erratum to:** *npj Microgravity* (2017) 3:6; doi: 10.1038/s41526-016-0003-7; Published 12 January 2017

In the original version of the article, Kevin Sato was incorrectly listed as an author instead of in the “Acknowledgements” section. The HTML and PDF version of the article has been corrected.

